# Open science practices in general and internal medicine journals, an observational study

**DOI:** 10.1371/journal.pone.0268993

**Published:** 2022-05-31

**Authors:** Beatriz Tarazona-Alvarez, Natalia Zamora-Martinez, Veronica Garcia-Sanz, Vanessa Paredes-Gallardo, Carlos Bellot-Arcis, Rut Lucas-Dominguez, Antonio Vidal-Infer

**Affiliations:** 1 Department of Stomatology, School of Medicine and Dentistry, University of Valencia, Valencia, Spain; 2 Department of History of Science and Information Science, School of Medicine and Dentistry, University of Valencia, Valencia, Spain; 3 UISYS, Joint Research Unit (CSIC – University of Valencia), Valencia, Spain; 4 CIBERONC, Valencia, Spain; University of Rennes 1, FRANCE

## Abstract

**Background:**

As part of the Open Science movement, this study aims to analyze the current state of open access and open data policies concerning the availability of articles and raw data of the journals belonging to the category “Medicine, General & Internal” of the Science Citation Index Expanded.

**Methods:**

Journal data sharing policies were evaluated through the following variables: possibility of manuscript storage in repositories; reuse policy; publication on a website; and statement regarding complementary material. Subsequently, an analysis of the supplementary material associated with each article was performed through the PubMed Central repository. The study reported was assessed following the STROBE guidelines for observational studies.

**Results:**

This study shows that only one-third of the journals included in the category “Medicine, General & Internal” allow the depositing of their documents in repositories and its reuse, while approximately half of the journals agree to publish the document on a website as well as to deposit supplementary material along with the publication. However, the reality about this last variable is that only 9.5% of the articles analyzed contained supplementary material being the main journals involved, *BMJ Open*, *JAMA Network Open*, *New England Journal of Medicine*, *Lancet and Plos Medicine*.

**Conclusions:**

The analysis of the opening policies of the journals concerning data availability in medical research reveals the unequal positioning of publishers towards the sharing of open data, the ambiguity regarding government policies about the obligation to deposit data and the need for ethical and standardization requirements in the typology/format of the data deposited without forgetting the important role that the researcher plays. Further studies based on journals indexed in medical databases other than Science Citation Index Expanded are needed.

## Introduction

Medical research affects all people, since all individuals are potential healthcare receivers; research outcomes in this field are therefore of interest for the whole population [1]. In this regard, open access editorial policies are recommendable to make publications available to both the scientific and the non-scientific population [2]. However, there are still several issues that make open access complicated and controversial to a point, such as article processing charges (APCs), which may condition the choice of authors to publish in one journal or another [3].

Open Science represents an scenario which includes not only open access, but also concepts such as open data, open source, open methodology or open peer review [4]. As part of the open research data, data sharing saves time, money and effort and can encourage new study designs, help avoid duplication, identify errors, promote research transparency, and reduce fraud [5, 6]. Recent studies have been made on the behavior of researchers referred to the sharing of raw data after completing and publishing their works. Different medical disciplines have been assessed, such as cell and tissue engineering [7], dentistry [8] and emergency care medicine [9]. The general conclusion of these studies is that while the sharing of data exists, it is scarce due to several reasons, such as the prohibition of different countries to share scientific information, the lack of incentives for researchers, and the lack of consensus regarding file and raw data format [10].

As a complementary way to promote transparency and get the most out of medical research, a number of options are available to allow data sharing. While some journals do not accept supplementary files, others place no restrictions [11]. Nevertheless, repositories are the best option, allowing data to be shared ethically and lawfully [5]. Different restriction levels can be found depending on the type of data sharing structure used—the following ranging from least to most restrictive: public archives, private archives, public enclaves and private enclaves [12]. In this context, public enclaves are structures for data sharing where any interested users may submit queries and receive aggregate results, and in private enclaves, only approved users may submit queries and receive aggregate results (often subject to review and approval of individual queries).

Based on previous studies, we hypothesize that results will show a lack of homogeneity and protocols for research data sharing. The aims of the present study were to: 1) Analyze the open access and open data policies concerning the availability of articles and raw data of the journals listed in the “Medicine, General & Internal” category of the Journal Citation Reports (JCR); 2) Assess the relationship between journal impact factor and the storage and reuse policies; and 3) Analyze the format of supplementary files regarding the quality of material that may be shared and reused.

## Methods

The report of the observational study was performed based on the STROBE guidelines, being the study units scientific articles. The methodology was developed in three steps following previous studies [7, 9], however the protocol was not registered. First, the instructions to authors published on the websites of the 165 journals included in the “Medicine, General & Internal” category of the 2019 Science Citation Index Edition of the JCR were reviewed. For each journal, policies regarding public availability of data sharing (where available) were documented and the following data were collected: (a) Journal name; (b) Publisher; (c) Journal website; (d) Information about the possibility of manuscript storage in thematic or institutional repositories, including the following variables: A: Allowed, when the manuscript can be deposited in institutional or thematic repositories and also when depositing in public repositories is required for publication in the journal; NA: Not Allowed, when explicit depositing in any repository is not allowed; NS: Not specified, when there is no clear information on depositing in a repository; (e) Reuse policy (A; NA; NS); (f) Policy regarding publication on the official website or by the author, such as publishing on their personal ResearchGate profile, etc. (N; NA; NS); and (g) Statement of policy regarding complementary material (A; NA; NS). Items (d), (e) and (f) refer to the availability of the article content, while (g) is the item related to the availability of raw data. This information was collected in September 2020. Journals were classified into quartiles according to the 2019 Science Citation Index Edition of the JCR.

As a second step, a subsequent search was performed in the PubMed Central (PMC) repository (the most widely used free full-text repository in biomedicine) to analyze the supplementary material associated with each article of each of the studied journals up to 31 July 2020. Of 165 journals indexed in the “Medicine, General & Internal” category of the Science Citation Index of the JCR, 146 are also present in PMC. Of the 408,899 articles in PMC, a total of 38,761 (9.5%) carried supplementary material. The research strategy used in PMC was designed to retrieve only articles with supplementary material: "journal name"[Journal] (<supplementary-material> or <supplemental-information>).

The third step consisted of a quantitative and qualitative analysis of supplementary information. The number and types of files located on the articles with supplementary material were registered even if a single article included several different files. In cases where there was a compressed file (e.g., zip or rar), it was opened to check the types of files it contained.

Data extraction was conducted manually by three different researchers and compared to agree on a consensus. All the information obtained was entered into an excel sheet, proceeding to its normalization and analysis. A descriptive analysis of the variables was carried out to obtain the frequencies and percentages.

## Results

**Fig 1:**
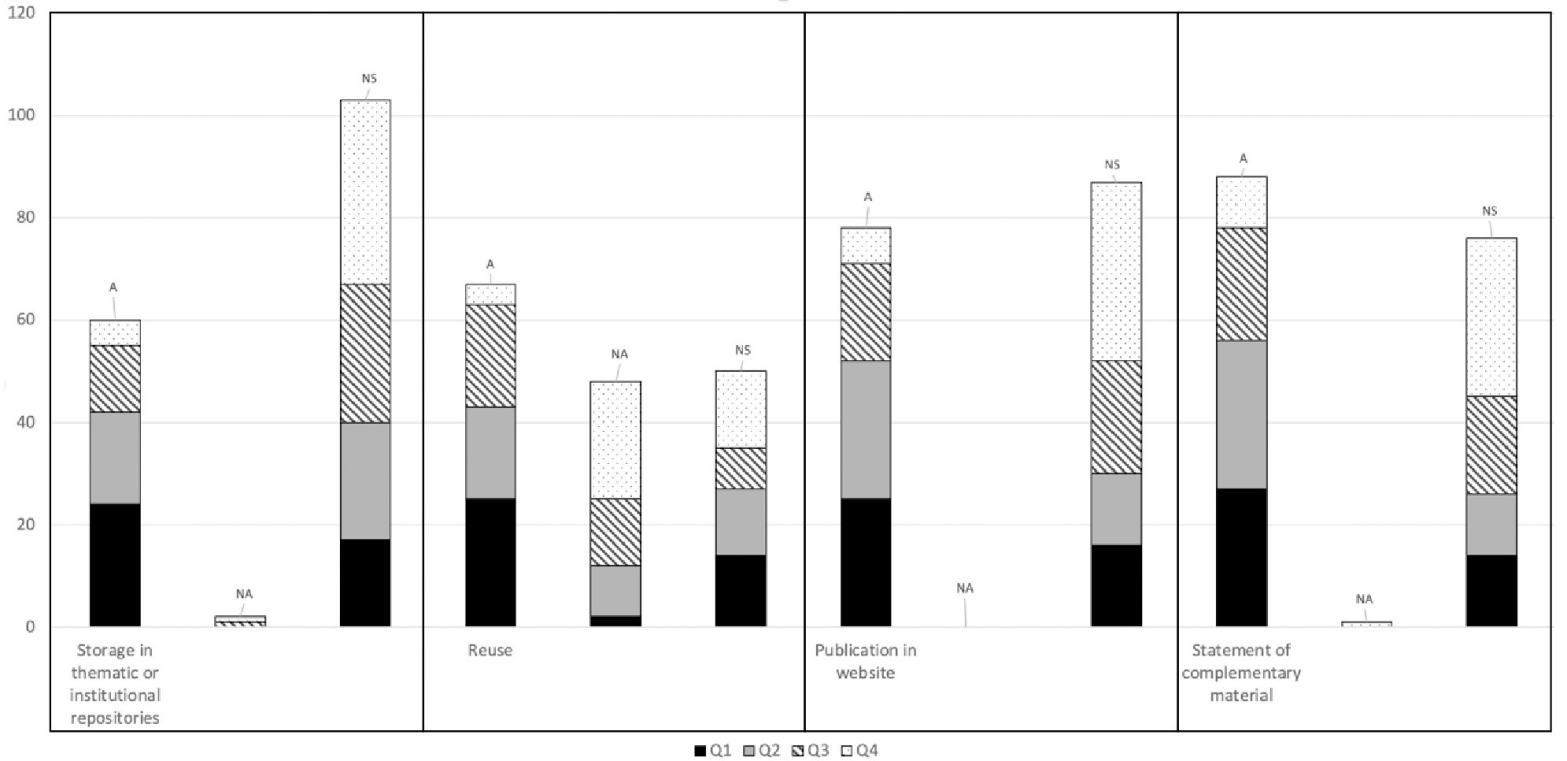
Key findings regarding open access and open data policies of the journals listed in the “Medicine, General & Internal” category.

Key findings regarding open access and open data policies of the 165 journals belonging to the “Medicine, General & Internal” category are represented in Fig 1. The results of the analysis of the main variables analyzed distributed by quartiles (Q) are shown in Table 1. With respect to the variable "Storage in thematic or institutional repositories", only 36.4% of the journals specified that storage is possible, while 62.4% did not specify such an option. The acceptance percentage was predominant in high impact journals (42 journals between quartiles 1 and 2) and was reduced by half in the case of journals in Q3 and Q4 (18 journals). Only in two journals belonging to Q3 and Q4, respectively, was this possibility denied.

**Table 1:** Analysis of variables concerning the availability of articles and raw data of the 165 journals, distributed by quartiles (Q). A: Allowed; NA: not Allowed; NS: not specified; T: Total.

	Storage in thematic or institutional repositories			Reuse			Publication on website			Statement of complementary material		
Q	A	NA	NS	A	NA	NS	A	NA	NS	A	NA	NS
1	24 (58.5%)	0 (0.0%)	17 (41.5%)	25 (61.0%)	2 (5.0%)	14 (34.2%)	25 (61.0%)	0 (0.0%)	16 (49.0%)	27 (65.9%)	0 (0.0%)	14 (34.2%)
2	18 (43.9%)	0 (0.0%)	23 (56.1%)	18 (43.9%)	10 (24.4%)	13 (31.7%)	27 (65.8%)	0 (0.0%)	14 (34.2%)	29 (70.7%)	0 (0.0%)	12 (29.2%)
3	13 (31.7%)	1 (0.0%)	27 (65.8%)	20 (48.7%)	13 (31.7%)	8 (18.5%)	19 (46.3%)	0 (0.0%)	22 (53.7%)	22 (53.6%)	0 (0.0%)	19 (46.3%)
4	5 (11.9%)	1 (0.0%)	36 (85.7%)	4 (9.5%)	23 (54.8%)	15 (35.7%)	7 (16.7%)	0 (0.0%)	35 (83.3%)	10 (23.8%)	1 (0.0%)	31 (73.8%)
**T**	60 (36.4%)	2 (1.2%)	103 (62.4%)	67 (40.6%)	48 (29.1%)	50 (30.3%)	78 (47.3%)	0 (0.0%)	87 (52.7%)	88 (53.3%)	1 (0.6%)	76 (46.1%)

Regarding the “reuse policy”, 40.6% of the journals allowed this possibility, with very similar behavior among the first three quartiles and with a notable decrease for Q4. In contrast, 29.1% of the journals do not allow reuse, while the remaining 30.3% failed to specify this possibility.

The variable “possibility of publishing the manuscript on a website” (Table 1) showed that 47.3% allowed this possibility (78 journals distributed mainly between Q1-Q3) and 52.7% (87 journals) failed to specify the possibility—the journals in Q4 being those showing least acceptance.

Lastly, the variable "statement of complementary material" was allowed in 53.3% of the cases (88 journals similarly between Q1-Q3, with journals in Q4 again being those showing least acceptance) while 46.1% of the journals failed to specify this option.

Of 165 journals indexed in the “Medicine, General & Internal” category of the Science Citation Index of the JCR, 146 were also present in PMC. Of the 408,899 articles in PMC, a total of 38,761 (9.5%) carried supplementary material, being the main journals involved *BMJ Open*, *JAMA Network Open*, *New England Journal of Medicine*, *Lancet* and *Plos Medicine* (Table 2).

**Table 2:** Metrics and journals including a percentage of supplementary material (SP) greater than 25%. All journals analyzed belong to the “Medicine, General & Internal” category of the 2019 Science Citation Index Edition of the JCR (S1 Table).

Quartile	Journal	Number of articles in PMC	Number of articles with SP in PMC	% of articles with SP in PMC
1	JAMA Network Open	1,907	1,388	72.8
1	New England Journal of Medicine	1,694	1,068	63.0
1	Lancet	1,161	580	50.0
1	PLOS Medicine	4,037	1,856	46.0
1	JAMA-Journal Of The American Medical Association	2,007	865	43.1
1	JAMA Internal Medicine	1,942	833	42.9
1	BMC Medicine	2,379	973	40.9
1	Annals of Internal Medicine	711	245	34.5
1	American Journal of Preventive Medicine	1,270	427	33.6
1	Journal of Cachexia Sarcopenia and Muscle	652	205	31.4
1	Palliative Medicine	188	52	27.7
1	Preventive Medicine	897	248	27.6
1	Journal of Clinical Medicine	3,275	893	27.3
2	BMJ Open	16,966	13,323	78.5
2	BMC Family Practice	2,176	879	40.4
2	Journal of Hospital Medicine	269	101	37.5
2	European Journal of General Practice	141	36	25.5
2	Internal and Emergency Medicine	20	5	25.0
3	Internal Medicine Journal	20	5	25.0

Table 3 shows the types of supplementary materials distributed according to the quartile of the journal in which it is published. A total of 54,958 files were from journals distributed in quartiles Q1 (57.4%), Q2 (34.7%), Q3 (7.8%) and Q4 (0.1%), respectively, taking into account the fact that a single article might contain several files (S1 Table).

**Table 3:** Quartile distribution of file formats of the supplementary material.

Quartile	Total	Pdf	Text	Image	Html	Spreadsheet	Media	Slideshow	Other
Q1	31,521 (57.4%)	11,759 (37.3%)	7,550 (24.0%)	3,493 (11.1%)	4,106 (13.0%)	2,425 (7.7%)	1,528 (4.8%)	402 (1.3%)	258 (0.8%)
Q2	19,076 (34.7%)	13,868 (72.7%)	2,860 (15.0%)	721 (3.8%)	239 (1.3%)	634 (3.3%)	102 (0.5%)	136 (0.7%)	516 (2.7%)
Q3	4,314 (7.8%)	1,111. (25.8%)	2,417 (56.0%)	218 (5.1%)	1 (0.0%)	223 (5.2%)	297 (6.9%)	24 (0.6%)	23 (0.5%)
Q4	47 (0.1%)	42 (89.4%)	5 (10.6%)	0 (0.0%)	0 (0.0%)	0 (0.0%)	0 (0.0%)	0 (0.0%)	0 (0.0%)

**Fig 2:**
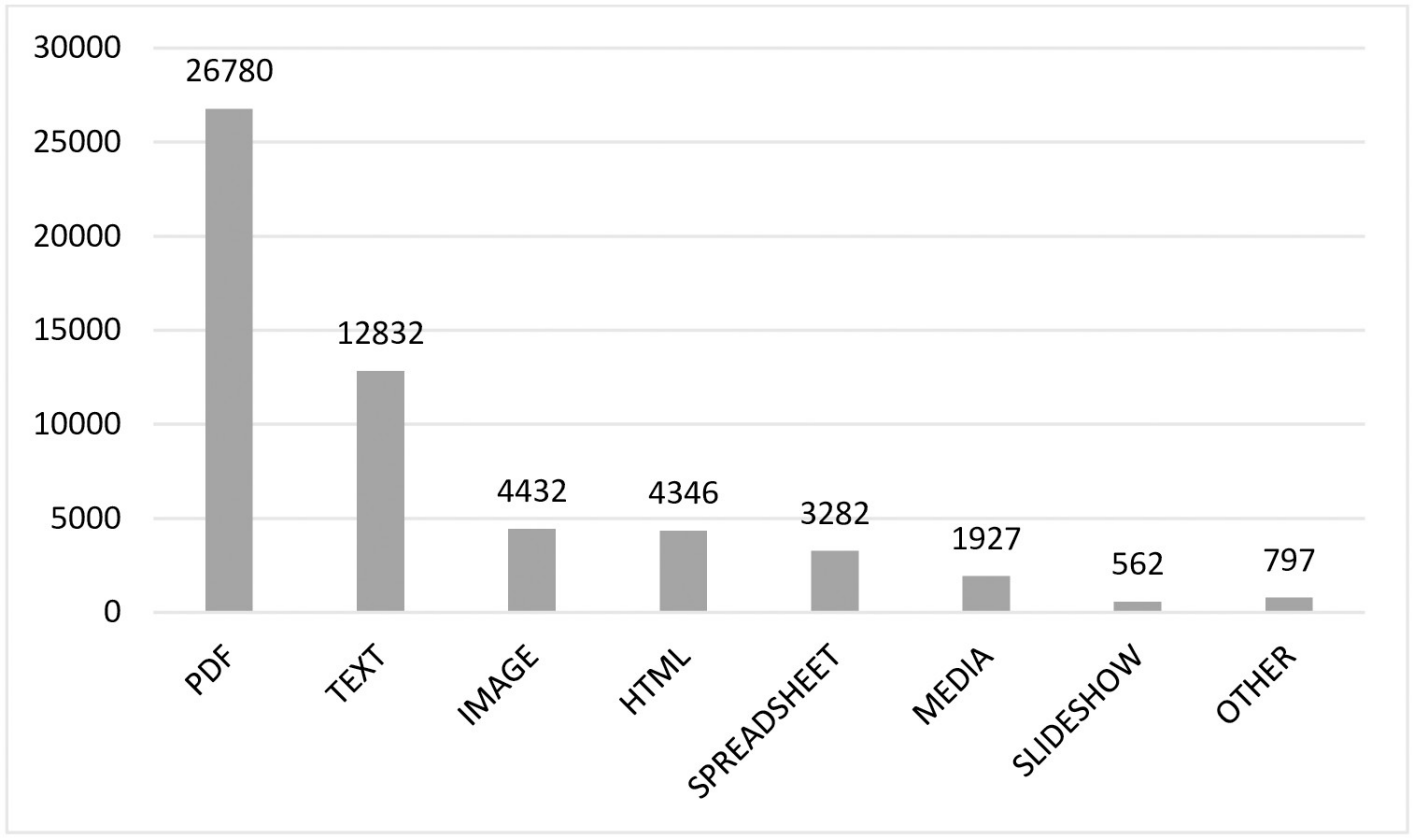
File formats of supplementary material (note that there may be more than one file per article).

The most frequent documents forming part of supplementary material were pdf (48.7%), text (23.3%), images (8.1%), html (7.9%) and spreadsheets (6.0%), containing information mostly referred to genes, molecular markers, and other results from analyzed substances and tissues (Fig 2).

## Discussion

The present study analyzed the current state of open access and open data policies of the journals belonging to the category “Medicine, General & Internal” of the Science Citation Index of the JCR and evaluated the types of data deposited by these journals through the PMC repository. Approximately one-third of the journals allow the depositing of their documents in repositories (60 journals) as well as reuse of the deposited material (67 journals). Half of the journals agree to publish the document on a website (78 journals) as well as include supplementary material along with the publication (88 journals). In all cases, these are journals mostly ranked in quartiles Q1 and Q2. Regarding the actual practice of depositing supplementary material together with the publication, the main journals involved belonged to Q1 and Q2 of the JCR.

The availability of data from medical research is a topic of growing interest worldwide. Its importance has recently been evidenced by the urgent need to share information to secure efficient and rapid responses in the past epidemics of the Zika and Ebola viruses and the current pandemic caused by the SARS-CoV-2 [9, 13], even though there may be some controversy regarding the transparency required in relation to sensitive data from clinical trials. However, the benefits are clear, since availability allows enriching information on diseases or their treatments in the shortest possible time, potentially improving the results of individual medical research, while promoting the reproducibility and sustainability of scientific activity [14, 15].

The category “Medicine, General & Internal” is an area in continuous evolution, where journals have improved their representation and impact on the Web of Science in recent years. The number of journals included has progressively increased over the last decade, from 100 journals in 2007 to 165 in 2019, covering medical specialties including general medicine and internal medicine, but also clinical physiology, pain management, military, and hospital medicine.

Our findings regarding open access policies show that, of the four variables analyzed, only the possibility of including supplementary material is allowed by more than half of the journals in this category (53.3%). This percentage is higher than the figures reported in other studies carried out in the category of Dentistry, without reaching the values obtained for Emergency Care Medicine [8, 9].

The secondary search through PMC revealed that only 9.5% of the articles contained supplementary material distributed in journals belonging to Q1 (57.4%), Q2 (34.7%), Q3 (7.8%) and Q4 (0.1%), respectively. Accordingly, the journals belonging to quartiles Q1 and Q2 contained the highest percentage of articles with supplementary material, which is justified by the responsibility acquired to maintain their quality and provide access to data supporting the published articles. This fact is further evidenced by the observation that the most representative typology (xls) of reusable raw data represents 7.7% in the articles published in Q1 journals, decreasing to 3.3% in Q2 or 5.2% in Q3 [16]. Examining the depositing of supplementary material, the data obtained for the category of “Medicine, General & Internal” (9.5%) coincide with those of previous publications in the categories of Emergency Care Medicine (9.4%) and Dentistry (7.6%). These results suggest that the sharing of open data in clinical and medicine research remains low [7–9].

The results obtained on the opening policies of the journals concerning data sharing in medical research revealed the unequal positioning in terms of the situation of journals towards the sharing of open data, the ambiguity regarding government policies on the obligation to deposit data and the lack of requirements for uniformity in the typology/format of the data deposited. In this regard, the FAIR principles [17] (findable, accessible, interoperable, reusable) have been established for data deposition in repositories; however, they should also be required for application to the raw data in supplementary material. Furthermore, a very important intervening factor must not be forgotten—the researcher—who should be motivated as well as trained for the practice of data sharing. Finally, and of utmost importance, technical and institutional barriers must be added especially in the “Medicine, General & Internal” category, due to sensitive healthcare information and patient data confidentiality [15, 18]. Hopefully, the unprecedented, accelerated and efficient open access and data sharing developed on occasion of COVID-19 crisis, exemplified when China publicly shared the genetic sequence of SARS-CoV-2 for the purpose of polymerase chain reaction diagnostic testing among other goals [13], will serve as a lesson to show that the benefits are enormous and that the drawbacks must be overcome through truly open platforms and regulatory policies for all stakeholders.

### Study limitations

This study has some limitations. Firstly, our methodology was based on previous published studies, but the protocol was not pre-registered. Secondly, we only analyzed medical journals included in JCR, and it is possible that there are other medical journals indexed in other databases with more flexible open data policies. However, the journals indexed in JCR remain those with the greatest impact internationally. Thirdly, only the PubMed Central repository has been searched since it is the main repository in health science today. Fourthly, the difference between both printed-online and completely online journals was not evaluated in this study, especially concerning the importance of sharing supplementary material. Finally, we do not know whether researchers actually reuse deposited published data.

## Conclusion

Approximately one-third of the journals allow the depositing of their documents in repositories (60 journals) as well as reuse of the deposited material (67 journals)—while approximately half of the journals agree to publish the document on a website (78 journals) as well as deposit supplementary material along with the publication (88 journals), especially in quartiles 1 and 2. Regarding the depositing of supplementary material together with the publication, 146 journals were in PMC with a total of 408,899 published articles, of which only 9.5% contained supplementary material (mostly pdf and doc files). The main journals involved in the depositing of supplemental material belonged to Q1 and Q2 of the JCR.

## Supporting information

S1 Table Metrics and journals including supplementary material classification sorted by quartile of the JCR “Medicine, General & Internal” category (2019 Science Citation Index Edition). (XLSX)
